# Accelerated aging of fast pyrolysis bio-oil: a new method based on carbonyl titration

**DOI:** 10.1039/d0ra00046a

**Published:** 2020-03-09

**Authors:** Stuart Black, Jack R. Ferrell

**Affiliations:** Biosciences Center, National Renewable Energy Laboratory Golden CO USA; National Bioenergy Center, National Renewable Energy Laboratory Golden CO USA Jack.Ferrell@nrel.gov

## Abstract

Fast pyrolysis bio-oils are known to age upon storage at room temperature, resulting in changes to both physical properties (increase in viscosity) and chemical composition (decrease in carbonyl content). A widely used accelerated aging test consists of holding samples at 80 °C for 24 hours, with viscosity measurement before and after heat treatment. Unfortunately, the viscosity measurement has high variability, and cannot be applied to samples that have phase separated. Here, we show that carbonyl content is a much better metric for tracking bio-oil aging. Furthermore, results from different accelerated aging protocols (for varying times at both 40 °C and 80 °C) are compared to actual room temperature storage for over 3 years. Based on this, we show that the accepted accelerated aging test (80 °C for 24 hours) is too severe a treatment, and results in more extensive aging than would occur with over 3 years of storage at room temperature. A new aging protocol is proposed: heat treatment at 80 °C for 2 hours, with carbonyl quantification before and after. This protocol correlates to room temperature storage for 1–3 months. Finally, samples were also kept in cold storage (at both 9 °C and −17 °C) for over 3 years. Unexpectedly, these samples also showed a substantial reduction in carbonyl content (by up to 25%), indicating that bio-oil aging still progresses at low temperatures. Both physical and chemical changes will occur in samples in cold storage, which has implications for the archiving of bio-oil samples.

## Abstract 2

Fast pyrolysis bio-oils are known to age upon storage at room temperature, resulting in changes to both physical properties (increase in viscosity) and chemical composition (decrease in carbonyl content).

## Graphical abstract

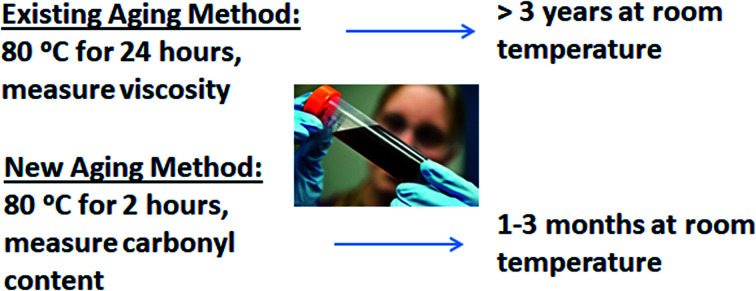

## Introduction

In recent years, the necessity of renewable energy technologies has grown, as the inevitable economic and environmental impacts of climate change have become widely accepted. At the same time, the cost of both wind and solar has significantly dropped, and the amount of renewable electricity generation has dramatically increased. Recently, it has been shown that energy sector emissions and economic growth have been decoupled, as energy-related CO_2_ emissions stayed flat in 2015 while the global economy grew.^1^ Considering this, it is obvious that renewable energy technologies are not only required to combat climate change, but can also lead to economic growth. Biomass will play a role, as it is a sustainable carbon feedstock which can replace petroleum feedstocks. Biomass conversion technologies must be developed to produce both liquid hydrocarbon fuels and renewable chemical precursors. The thermochemical deconstruction of biomass *via* fast pyrolysis is a promising pathway, which produces a liquid product (bio-oil) that can be further processed to fuels and chemicals.

Fast pyrolysis (FP) can produce bio-oil in high yields of up to 70 wt% of the dry biomass feed. In addition, bio-oil is an energy-dense liquid which can be stored and transported. FP bio-oil is currently being investigated for multiple upgrading pathways to produce fuels and chemicals, including hydrotreating^2^ as well as co-processing in a petroleum refineries.^3^ Catalytic fast pyrolysis^4^ (CFP) is also an area of active research. FP bio-oils are significantly different from petroleum crude oils, and therefore existing petroleum technologies may not apply to processing bio-oils. A typical FP bio-oil is an acidic liquid (pH *ca.* 2.5), contains large amounts of water (20–30 wt%), and has a high oxygen content (*ca.* 50 wt%). Detailed analyses have shown that FP bio-oil contains more than 300 compounds,^5^ and that oxygen exists in a variety of chemical functionalities, including acids, alcohols, aldehydes, ketones, esters, ethers, phenolics, sugars, and furans. Due to this complex chemical composition, detailed and reliable analysis of bio-oil remains a challenge, though some standardized chemical characterization methods have recently been published.^6^

Furthermore, FP bio-oils are highly reactive. They contain organic acids, phenolic compounds and aldehydes; the presence of all three of these constituents provides the necessary components for the formation of a phenol/formaldehyde resin, similar to a resol^7^ resin. The condensation reactions of the aging process^8^ cause an increase in both molecular weight and viscosity of the bio-oils over time, and viscosity has traditionally been used to indicate aging of the oils. Viscosity is a logical physical property for gauging bio-oil aging, as an increase in viscosity could directly affect processing. However, the viscosity measurement is prone to high variability.^9^ Some FP bio-oils are more reactive than others and can phase separate during the aging process. Homogenizing the samples following phase separation can be difficult, making it impossible to obtain a representative sample suitable for viscosity measurement. Measuring viscosity of a phase separated bio-oil results in highly variable measurements and erroneous results.^10^ Practically, this is a significant problem for using viscosity to determine how much a bio-oil has aged. Further, the times and temperatures used to age the oils are restricted by the phase separation problem if viscosity is used to track aging. The commonly used aging protocol heats samples at 80 °C for 24 hours,^9,11–13^ however at these conditions many bio-oil samples will phase separate. The reactions that form the condensation products can have a widely varied effect on the viscosity of the sample as well. While the formation of the condensation products increases the viscosity of the bio-oil, formation of water from the condensation decreases the viscosity.

In recent years, different aspects of bio-oil aging have been studied at length. There has been interest in the phase stability of FP bio-oils,^14^ including approaches to controlling the phase stability based on solvent fractionation^15^ or employing specific additives,^16^ and performing accelerating aging on the produced fractions.^17,18^ Furthermore, the phase stability of FP bio-oils has recently been reviewed.^19^ The chemical composition of aged bio-oils has been reported,^20–25^ and other advanced characterizations have been performed.^26^ It has also been shown that bio-oil aging is an exothermic process.^27^ Despite many in-depth studies, the accepted accelerated aging test has remained the same: a 24 hour heat treatment at 80 °C, with measurement of viscosity before and after; it has been stated that this aging test corresponds to 6–12 months of storage at room temperature.^9^ Despite poor results in recent validation activities,^9^ this aging test remains in use today.

It has previously been shown that aging can be tracked using carbonyl content,^28^ as carbonyl content decreases as the bio-oil sample ages. As the resol reactions proceed, condensation reactions between phenolic moieties and aldehydes form methylene structures, increasing molecular weight; carbonyl content decreases as the aldehydes are consumed. Although carbonyl titration has been used for FP bio-oils for several decades, Black and Ferrell^29–31^ recently developed a new carbonyl titration method that substantially improves the previous method by decreasing reaction time while increasing both accuracy and precision. Additionally, this new carbonyl titration method has been approved as a ASTM Standard Test Method E3146,^32^ which is the first ASTM method solely focused on chemical characterization of pyrolysis bio-oils. In contrast to the viscosity measurement, carbonyl titration can be applied to samples that have phase separated. The new aging test presented here, based on Black/Ferrell carbonyl titration method, provides more reliable results with less variance and less interference from competing products, as compared to the traditional aging test based on viscosity measurement.

In this paper, we have performed accelerated aging on many different FP bio-oils produced from different feedstocks and at different pyrolysis conditions. Similar aging behavior was seen across this broad suite of different bio-oils, therefore the conclusions drawn apply generally to FP bio-oils. Here, we will also demonstrate that the common bio-oil aging method consisting of heating the oil at 80 °C for 24 hours is too harsh to accurately represent aging at room temperature. With accelerated aging at 80 °C, resol reactions are driven to completion too quickly, often before 24 hours. Comparisons will be made of accelerated aging protocols at different temperatures (40 °C and 80 °C) with actual aging of samples held at room temperature for long periods of time (up to 3.4 years). Finally, a new protocol for accelerated aging of FP bio-oils based on carbonyl titration will be presented.

## Experimental

Samples (50 g) of 9 FP bio-oils from 3 different feedstocks (Table 1: Oak, Pine and Blend 1) and 3 different conditions (Table 2) were aged at 19 °C (standard deviation = 0.4 °C) for 3.4 years (29 352 hours) in sealed 100 mL Schott bottles. Samples were taken periodically (Table 3) over a time span of 3.4 years.

**Table 1:** Biomass feedstocks and feedstock blends used to produce bio-oil samples. FR: forestry residues; acFR: air-classified forestry residues; C & D waste: construction & demolition waste; SG: switchgrass; poplar: hybrid poplar

Name	Sample designation	Source	Oak%	Pine%	FR%	acFR%	C & D waste%	SG%	Poplar%
Oak	Oak	Country Boy	100						
Clean pine	Pine	INL		100					
Blend 1	Blend1	INL		30	35		25	10	
Blend 2	Blend2	INL		45	25		30		
Blend 3	Blend3	INL		30		60			10

**Table 2:** Pyrolysis process conditions

Condition	01	02	03
Pyrolysis temperature	500 °C	480 °C	500 °C
Residence time	4 s	4 s	3 s

**Table 3:** Bio-oil sampling times at each storage and aging temperature for Blend1, Oak, and Pine bio-oil samples

Temperatures & sampling times (h = hours)				
19 °C	−17 °C	9 °C	40 °C	80 °C
144 h			2 h	2 h
672 h			6 h	6 h
2256 h			8 h	10 h
4368 h	4368 h		10 h	24 h
6528 h	7056 h	6624 h	24 h	36 h
8832 h	8832 h	8832 h	48 h	48 h
10 944 h	10 944 h	10 944 h	72 h	72 h
13 392 h	13 392 h	13 392 h	168 h	120 h
17 616 h	17 616 h	17 616 h		
29 352 h	29 352 h	29 352 h		

Samples (5 g) of the same bio-oils were heated in a Blue Line oven (Model SW-11TA-1) at 40 °C for 7 days (168 h) and 80 °C for up to 5 days (120 h) in sealed 10 mL Schott bottles. Samples of the original bio-oils were stored in a −17 °C (standard deviation = 5.1 °C) frost-free freezer for 3.4 years, with samples taken for carbonyl quantification at 6, 9, 12, 15, 18, 24, and 40 months. Additional samples were stored in a 9 °C (standard deviation = 0.6 °C) cold room for 40 months, with samples taken at 9, 12, 15, 18, 24, and 40 months for carbonyl analysis. Complete details of sampling times and storage temperatures are shown in Table 3.

Additional samples from several other feedstocks (Blend 2, Blend 3) were aged at 80 °C but without the room temperature aging nor the 40 °C aging. Bio-oil samples used for this study were produced *via* fast pyrolysis from commercially available oak feedstock (Country Boy White Lightning Heating Pellets) and also from feedstocks and custom feedstock blends (see Table 1) provided by Idaho National Laboratory (INL). Bio-oil was produced in the National Renewable Energy Laboratory's (NREL) Thermal and Catalytic Process Development Unit (TCPDU), a half-ton per day, pilot-scale plant used to research thermochemical routes of producing liquid fuels from biomass.

To produce bio-oil in the TCPDU, first the biomass was crushed to less than 2 mm, entrained in preheated nitrogen gas, and then fed into the entrained-flow pyrolysis reactor at conditions presented in Table 2. Char and ash were removed from the pyrolysis vapors by cyclones, and the vapors were then condensed into bio-oil in a liquid scrubber using dodecane as the scrubbing liquid. The bio-oil was filtered through a 10 μm liquid filter and separated from the dodecane in a phase separator. The bio-oil was then drained off the bottom of the phase separator and the dodecane was recirculated back to the scrubber. The bio-oil produced during these runs appeared to be one phase of uniform oil; no aqueous phase was observed during production. Additional details about the TCPDU at NREL can be found in the following ref. 33. As bio-oil samples were made using both different feedstocks and different pyrolysis conditions, samples are named as follows: feedstock_pyrolysis condition. For example, Blend1_02 designates a bio-oil made from Blend1 feedstock (described in Table 1) at pyrolysis condition 02 (described in Table 2).

Bio-oils produced in the TCPDU were analyzed for C, H, N, O content, where O content was calculated by difference (Huffman Hazen Laboratories). Water content was measured by Karl Fischer titration (Huffman Hazen Laboratories). Acid titration, providing data on the carboxylic acid number (CAN) and total acid number (TAN) was performed in-house, following a method described previously.^34^ Similarly, insoluble solids were measured in-house following ASTM D7579.

Carbonyl titration was performed using the Black/Ferrell method.^29–31^ This method has been shown to be very reliable, with a variability of 5% relative standard deviation (RSD) for typical FP bio-oils.^6^ Additionally, this method employs validation with a known carbonyl compound to ensure proper operation of titration equipment. Measurement of dynamic viscosity was performed in a Brookfield viscometer (Model VD2T) fitted with a Wells-Brookfield cone and plate cup with a CPA40Z spindle. The temperature was controlled at 40 °C with a Lauda (Model K2/RD) recirculating bath. The Wells-Brookfield cone and plate cup allows for a small sample size. A sample of 0.5 mL of oil was drawn up into a 1 mL syringe, weighed and added to the cup. The sample was allowed to equilibrate, and the viscosity was measured for 15 minutes with the average being taken over the last 10 minutes of the experiment. Samples that had phase separated were homogenized by heating in a 50 °C oven for 15 minutes, as well as agitated to ensure complete mixing of the two-phase samples, prior to sampling. Viscosity was not measured on samples that could not be homogenized. All measurements were performed in triplicate, and error bars were calculated from the standard deviation.

## Results & discussion

Data on elemental analysis, water content, carboxylic acid titration (providing a carboxylic acid number (CAN) and a total acid number (TAN)), and insoluble solids can be seen in Table 4. These routine characterizations show that the FP bio-oils tested exhibited chemical and physical characteristics typical of FP bio-oils. For example, water contents ranged from 20–31% depending on the feedstock used. Additionally, all bio-oils had oxygen contents near 50%. The Oak bio-oil showed slightly lower water and oxygen contents, as well as higher CAN and TAN values, than the bio-oils produced from pine and blended feedstocks.

**Table 4:** Properties of FP bio-oils. CAN & TAN units are mg KOH per g

	Water (wt%)	Carbon (wt%)	Hydrogen (wt%)	Nitrogen (wt%)	Oxygen (wt%)	CAN	TAN	Insoluble solids (wt%)
Pine	22.4	43.00	7.66	0.15	49.2	65.9	155.4	0.028
Oak	19.9	44.04	7.39	0.09	48.5	90.9	164.0	0.032
Blend1	31.0	38.88	8.04	0.32	52.7	67.2	149.8	0.005
Blend2	27.1	40.23	7.67	0.16	51.9	67.6	151.5	0.039

**Fig. 1:**
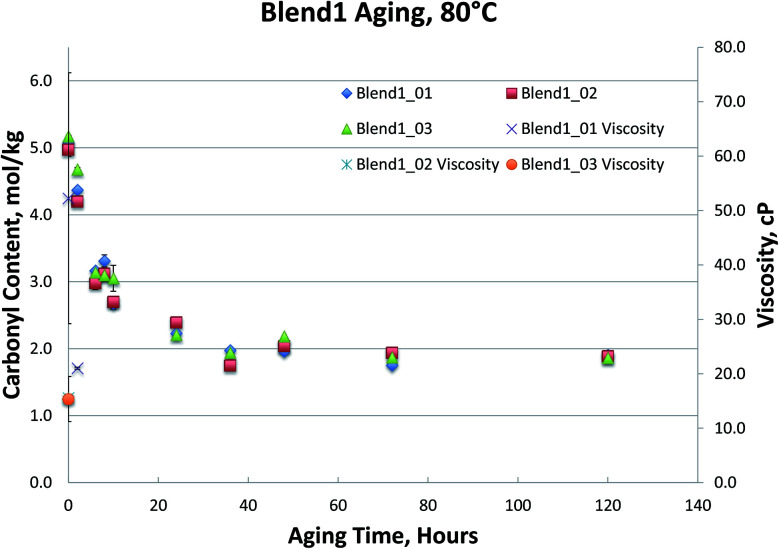
Blend1 bio-oils 80 °C aging.

**Fig. 2:**
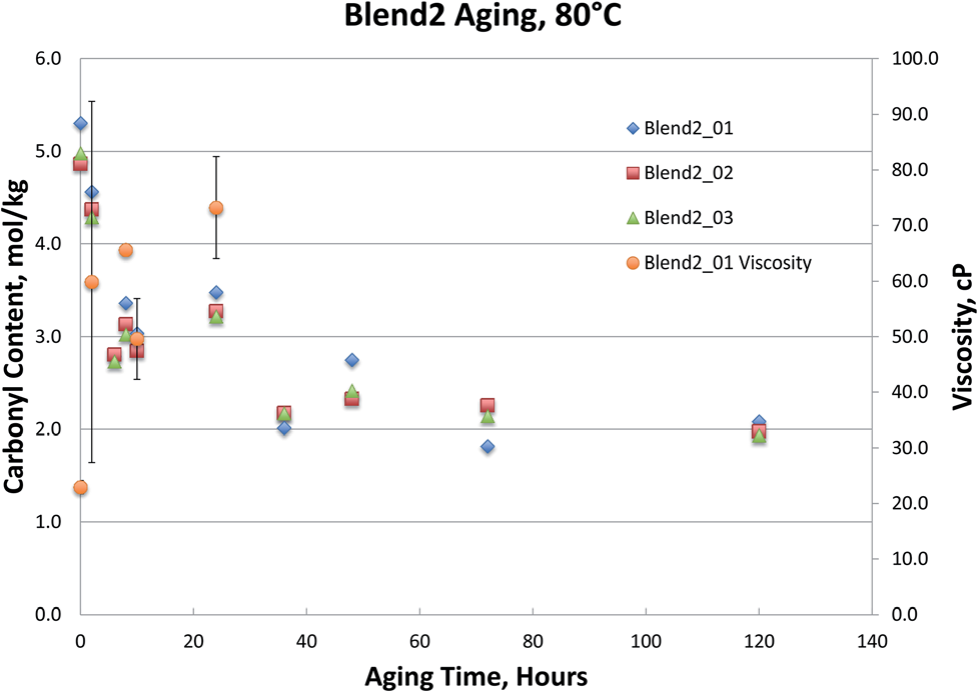
Blend2 bio-oils 80 °C aging.

**Fig. 3:**
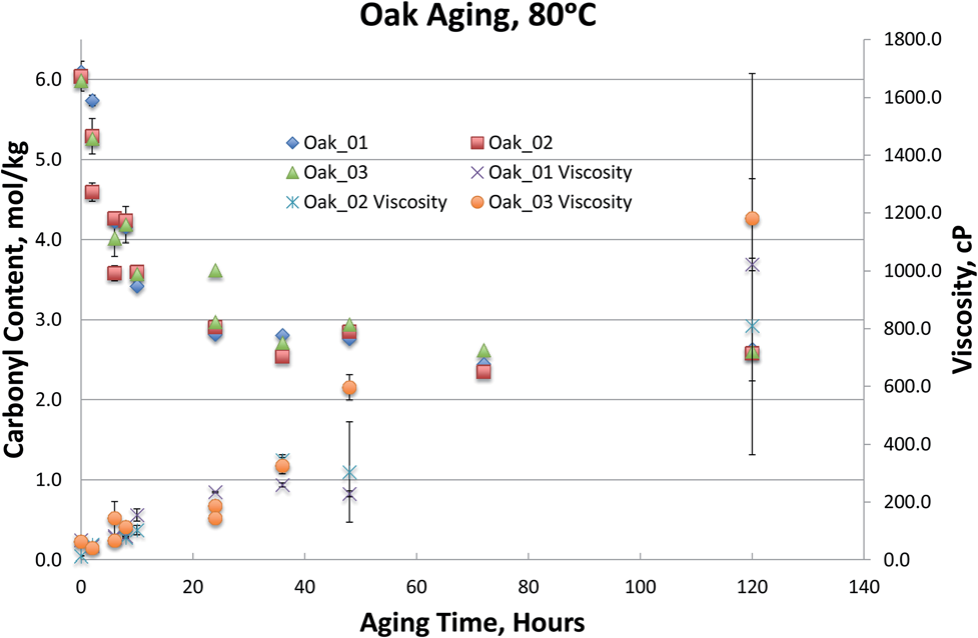
Oak bio-oils 80 °C aging.

**Fig. 4:**
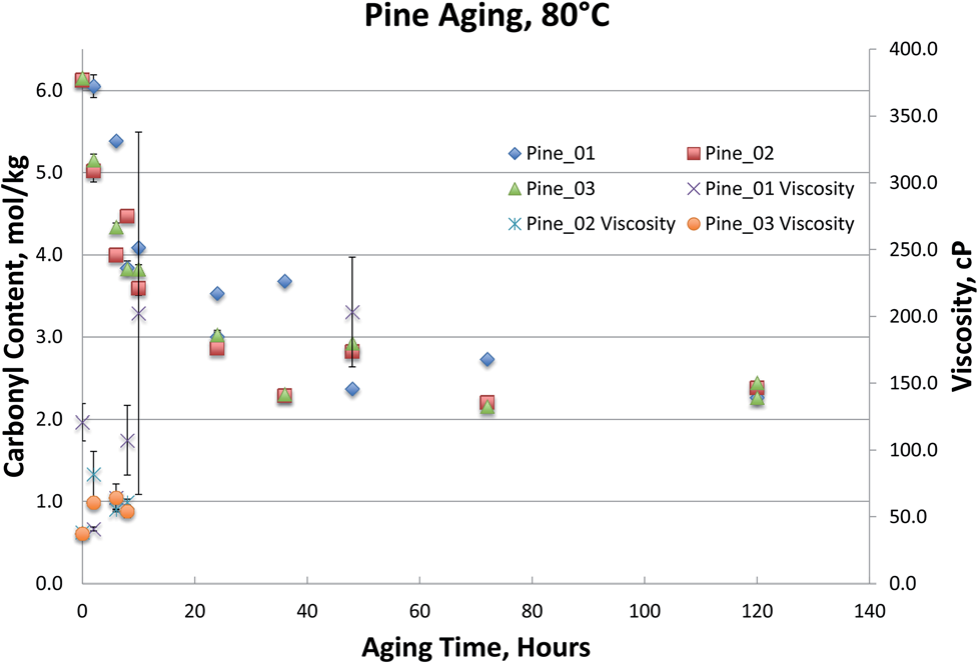
Pine bio-oils 80 °C aging.

Fig. 1–4, for Blend1, Blend2, Oak, and Pine bio-oils respectively, show a comparison of bio-oils aged at 80 °C for times varying from 2 hours to 120 hours and then analyzed by carbonyl titration and viscosity. Prior to aging, all bio-oils show carbonyl contents in the 5–6 mol kg^−1^ range, with Oak and Pine bio-oils having higher carbonyl contents near 6 mol kg^−1^, and Blend1 and Blend2 bio-oils having carbonyl contents near 5 mol kg^−1^. For each feedstock, multiple pyrolysis conditions (condition 01, 02, or 03, defined in Table 2) are shown. Fig. 1–4 show similar trends, where carbonyl content decreases and viscosity increases with aging time. This confirms previous findings showing that carbonyl content can be used to track bio-oil aging, as carbonyls reliably decrease upon aging.^28^ For all bio-oils studied, a rapid decrease in carbonyl content is seen during the first 10 hours of accelerated aging at 80 °C. By *ca.* 20 hours of aging, carbonyl contents are about half of the fresh (non-aged) bio-oils. The carbonyl contents reached stable values upon long aging times for all bio-oils. For both Blend1 and Blend2 samples, stable carbonyl contents of 2 mol kg^−1^ were achieved by 40 hours of aging, and further aging did not further decrease carbonyl content. For Oak and Pine bio-oils, stable carbonyl contents were slightly higher at *ca.* 2.5 and 2.3 mol kg^−1^, respectively.

Nearly all samples except the Oak bio-oil phase separated at some point during the aging. Most notably, Blend2 samples phase separated within 2 to 6 hours at 80 °C, and the Blend1 bio-oils were phase separated upon receipt for analysis. This phase separation behavior is expected and follows the water content of these bio-oils, where samples with more water are more prone to phase separation. While both Blend1 and Blend2 are mainly comprised of clean pine, forestry residues, and construction & demolition waste, Blend1 also contains 10% switchgrass. Correspondingly, Blend1 bio-oil had higher water content than did Blend2, and was more prone to phase separation.

As shown in Fig. 1 and 2, viscosity is unable to track aging for both Blend 1 and Blend 2 samples due to phase separation. As mentioned above, phase separation prevents the reliable measurement of viscosity, and in Blend 1 and Blend 2 samples, only the Blend2_01 sample was still single-phase after 2 hours at 80 °C. Even when samples that did not phase separate and viscosity could be measured, the viscosity measurement exhibited high errors. Due to both high variability and an incomplete data set (due to phase separation), no conclusions can be drawn from the viscosity data on the aging of the Blend1 and Blend2 oils. The unreliability of the viscosity measurement for bio-oils has been well documented in past studies.^9^ In fact, in a previous round robin study on the viscosity-based stability test (accelerated aging for 24 hours at 80 °C), it was found that viscosity could only track aging for samples where insoluble solids had been removed. Even with insoluble solids removed, the variability of this aging test was still found to be too high. Based on viscosity measurement, the authors stated that accelerated aging for 24 hours at 80 °C corresponded to 6–12 months of storage at room temperature.^9^ In the present study, insoluble solids were not removed, and were present in all samples under investigation (see Table 4). In addition to the phase separation issue which led to an incomplete data set, high variability was seen from viscosity measurement, and is attributed in part to the presence of insoluble solids.

The carbonyl measurement, however, can be performed on samples that have phase separated, and exhibits significantly lower errors than viscosity. All measurements were performed in triplicate, and error bars (taken as the standard deviation) are included in Fig. 1–4; these errors are very low, typically below 5% RSD, which has been shown previously in the inter-laboratory validation of this carbonyl titration method.^6^ Of all the samples analyzed, the Oak oil was the only sample to remain single phase throughout aging at 80 °C. All three 72 hour Oak samples were too thick for viscosity measurement and returned a viscosity in excess of 3000 cP, which is the limit for the size of spindle used in the viscometer. As shown in Fig. 3, the viscosity for aged samples up to 48 hours is reproducible but the viscosity for the 120 hour sample had very high variability. Neglecting the 120 hour sample, a straight-line fit can be made to the viscosity up to 48 hours.

Viscosity of the Oak_02 sample at 48 hours is more highly variable then the other oak samples. This is due to both a content of insoluble solids in the sample and to the nature of the viscosity measurement. There is a very small gap between the cup of the viscometer and the rotor (by design). Particulates can jam the spindle and return an erroneous reading. This may be the reason that all the 72 hour samples were so much higher in viscosity and could also be the reason for the high variability of the 120 hour samples. Given that pyrolysis bio-oils contain insoluble solids, this is an inherent limitation of the viscosity measurement.

The Pine samples suffered from phase separation after 10 hours at 80 °C with the exception of the Pine_01 sample. Again, however, particulate content was a problem with the samples and caused high variability in the viscosity measurement. As with the Oak, Blend1, and Blend2 samples, the carbonyl titration is shown to be a much more reliable measurement than viscosity. Further, viscosity measurement was not possible for samples that had phase separated, which is a common occurrence upon bio-oil aging.

**Fig. 5:**
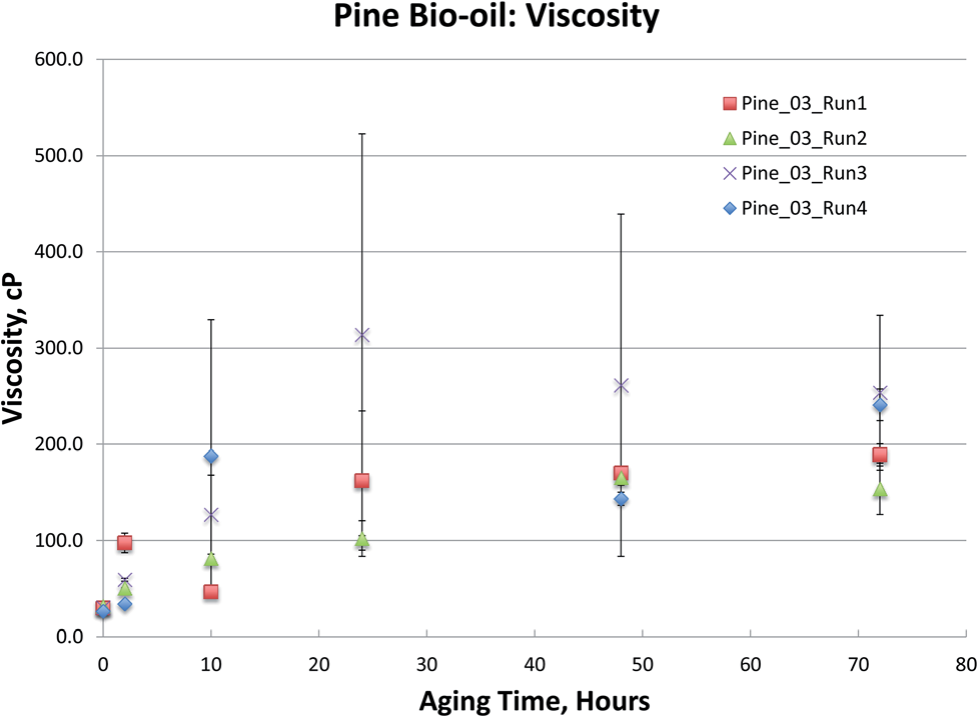
Viscosity of Pine bio-oils aged at 80 °C.

To further compare the utility of carbonyl titration *versus* viscosity measurement for tracking bio-oil aging, several Pine bio-oils were aged at 80 °C, followed by both viscosity and carbonyl measurement. Samples were removed from the oven after 2, 10, 24, 48 and 72 hours of aging at 80 °C. The samples phase separated after 24 hours but could be homogenized by gently heating them as detailed in the experimental section. Each sample was analyzed in triplicate and the variances are shown in Fig. 5 for the viscosity measurement. While all Pine bio-oils in Fig. 5 were produced at the same pyrolysis condition, they were produced during different production runs that occurred on separate days. Fig. 5 shows a trend for each oil that appears to be linear, however, there are large errors for several samples. Given the error in these measurements, it is not clear what type of curve would provide the best fit to the data. The magnitude of the errors effectively prevents the use of the viscosity measurement for tracking bio-oil aging processes.

**Fig. 6:**
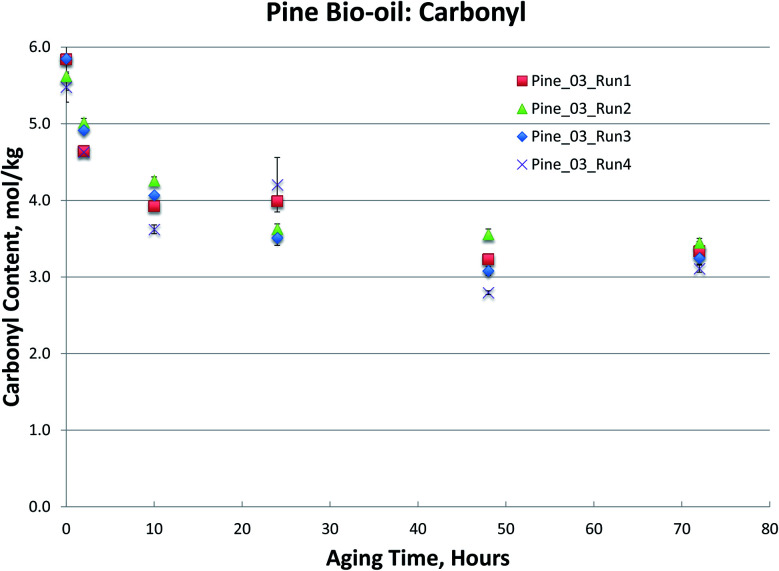
Carbonyl content of Pine bio-oils aged at 80 °C.

Carbonyl titration of the same samples again exhibits very small errors. As the sampling interval during accelerated aging was increased in Fig. 6, it more clearly shows that carbonyl content decreases exponentially during accelerated aging. More importantly, the carbonyl measurement is very reproducible, and a clear trend can be seen; this was not the case for viscosity measurements. As with the Oak, Blend1, and Blend2 samples, the carbonyl titration is shown to be a much more reliable measurement than viscosity. For all bio-oils studied, carbonyl measurement consistently exhibited smaller errors than the viscosity measurement. Further, viscosity measurement was not possible for samples that had phase separated, which is a common occurrence upon bio-oil aging. For these reasons, we propose that carbonyl titration be used in place of the viscosity measurement as a metric to track bio-oil aging, and only carbonyl titration results will be presented for the remainder of this manuscript.

### Accelerated aging *vs.* long-term storage

**Fig. 7:**
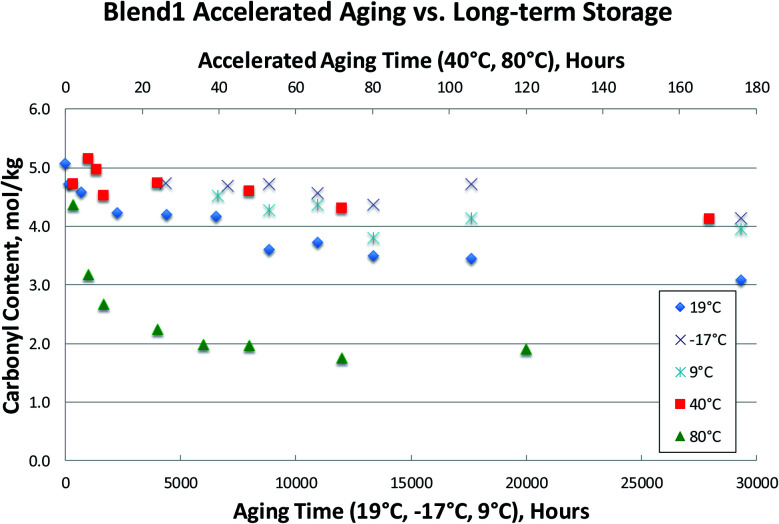
Accelerated aging *vs.* long-term storage: carbonyl contents for Blend1_01 bio-oil.

**Fig. 8:**
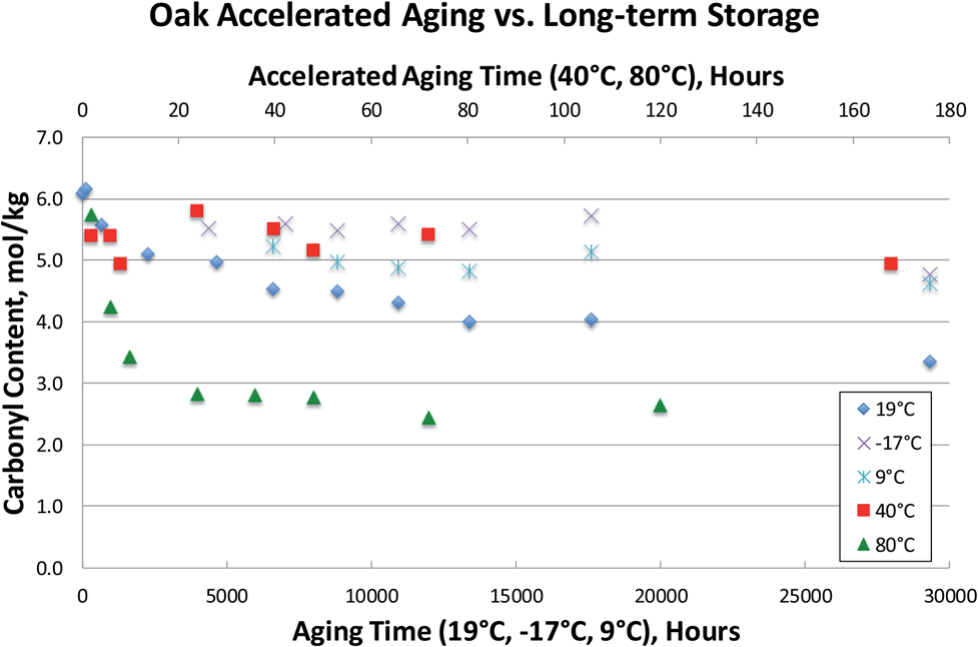
Accelerated aging *vs.* long-term storage: carbonyl contents for Oak_01 Bio-oil.

**Fig. 9:**
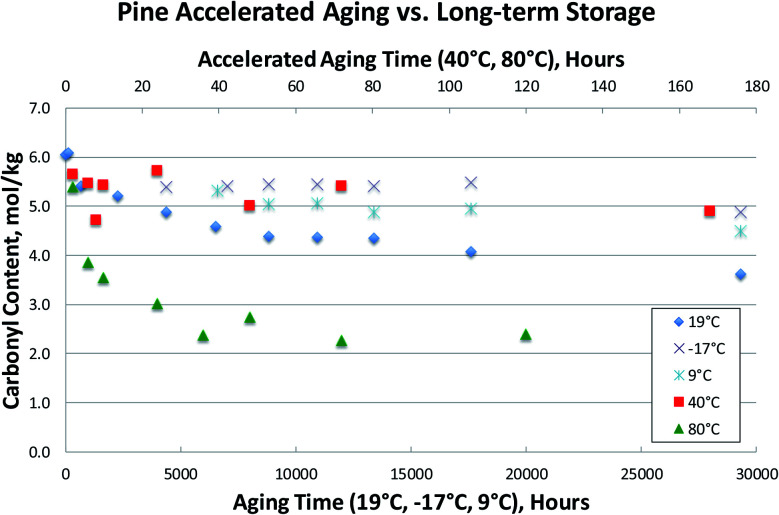
Accelerated aging *vs.* long-term storage: carbonyl contents for Pine_01 bio-oil.

Several bio-oil samples were subjected to accelerated aging at two different temperatures (40 °C and 80 °C) as well as long-term storage at room temperature and at two different cold storage conditions (9 °C and −17 °C). Only the carbonyl contents were measured due to the poor viscosity results as previously discussed. This data is presented in Fig. 7–9, where accelerated aging at elevated temperatures maps to the top horizontal axis, and long-term storage both at room temperature and in cold storage conditions maps to the bottom horizontal axis.

Fig. 7–9 illustrate that the commonly used accelerated aging protocol of holding at 80 °C for 24 hours is far too harsh, and ages the samples more than would occur in over 3 years at room temperature. As can be seen in Fig. 7–9, room temperature storage (at 19 °C) of bio-oils for 3.4 years have aged less (having higher carbonyl contents) than samples subjected to accelerated aging for 24 hours at 80 °C. These results show that 80 °C rapidly promotes bio-oil aging, resulting in extensive condensation of the aldehydes and phenols in the bio-oil in a matter of hours. Just 12 hours of accelerated aging at 80 °C correlates to over 3 years of room temperature storage for the Blend1, Oak, and Pine bio-oils studied. For Blend1, Oak, and Pine samples 2 hours at 80 °C correlates to between 1–3 months or room temperature storage.

Meng^20^ presented an aging index for pyrolysis oils:Aging index = (*P*_Initial_ bio-oil − *P*_Aged_ bio-oil)/*P*_Initial_ bio-oil × 100%

Using the aging index, room temperature aging results in a reduction of the carbonyl content of *ca.* 20–25% after 9 months of aging at room temperature. The reduction of the carbonyl content at 40 °C after 170 hours is *ca.* the same 20–25% reduction. But accelerated aging at 80 °C results in a reduction of >50% of the original carbonyl content in 24 hours. This correlates to more aging than was seen for 3.4 years at room temperature, and results in the complete consumption of reactive carbonyls as can be seen by the flattening of the curve after 24 hours at 80 °C.

While an increase in viscosity due to room temperature storage will eventually impede bio-oil processing, it is possible that some amount of aging could be advantageous for certain processes. For example, it has been shown that total carbonyl content can be used to predict performance during thermocatalytic upgrading. Here, it was found that the high temperature hydrotreatment reactor would plug during processing of FP bio-oils with too high of a carbonyl content.^35^ In this processing scheme, lowering of the carbonyl content *via* some amount of room temperature aging could be desirable. For the emerging heating applications (use of bio-oil in industrial burners), storage at room temperature seems likely, at least for shorter periods of time (*e.g.*, weeks). For the production of fuels and chemicals *via* upgrading technologies, storage of bio-oils at room temperature may still be likely depending on the location of bio-oil production and upgrading facilities, and as discussed above, some room temperature storage could be advantageous. To determine how much a bio-oil sample will age upon prolonged storage at room temperature, an accelerated aging test must be performed. As shown above in Fig. 7–9, carbonyl content can be used to accurately and reliably track aging at room temperature. We have also shown that the commonly used accelerated aging protocol (80 °C for 24 hours, with viscosity measurement before and after heat treatment) is too severe, resulting in more aging than will occur at room temperature. Additionally, this protocol is unreliable due to variability of the viscosity measurement, and the fact that viscosity cannot be measured on phase-separated samples.

Therefore, we propose a new accelerated aging protocol: 80 °C for 2 hours, with carbonyl measurement before and after heat treatment. While aging at 40 °C more closely tracks the long-term room temperature aging in all cases, execution of accelerated aging at 40 °C would take up to 120 hours. This is too long to be practical in an industrial setting. Therefore, accelerated aging for 2 hours at 80 °C would correlate to a more reasonable amount of room temperature storage of between 1–3 months, depending on the bio-oil sample. It is worth mentioning that 2 hours has been chosen as it correlates to realistic amounts of time a bio-oil would be stored. However, as seen in all 80 °C accelerating aging results, it is clear that the sample rapidly ages for the first several hours, and that this aging comes to completion by *ca.* 24 hours. To more clearly understand how bio-oils age, data is presented below where aging at 80 °C is performed in 15 minute increments for the first 4 hours of aging. This experimental protocol can be used to correlate to shorter amounts of room temperature. Additionally, shorter and more frequent heat treatments can be used to determine when bio-oil aging has come to completion, that is, when the reactive carbonyls have been depleted, as discussed below.

### Accelerated aging at short times

**Fig. 10:**
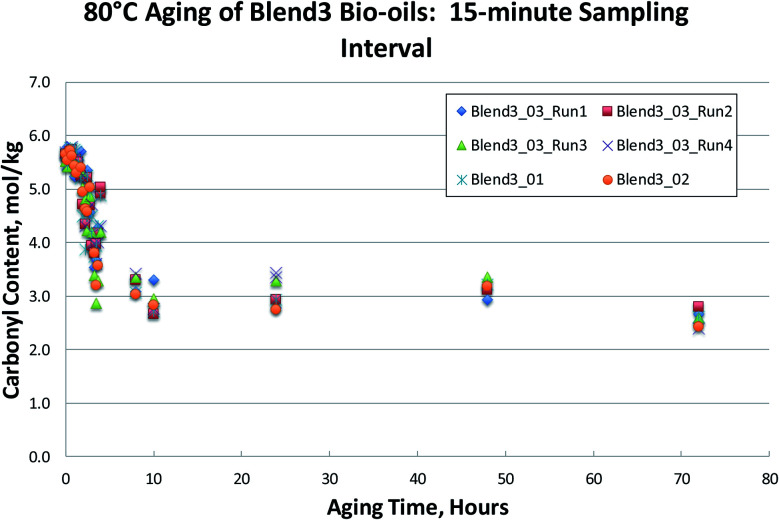
Fifteen-minute sampling of Blend3 bio-oils aged at 80 °C.

To test shorter 80 °C accelerated aging times, a new set of fresh bio-oil samples was obtained. Six different oils produced from a pine-based feedstock blend (Blend 3) under different conditions were aged for up to 72 hours. Samples were taken at 15 minute intervals for the first 4 hours and carbonyl content was analyzed by the Black/Ferrell method. The results are shown in Fig. 10. These frequent heat treatments, for a variety of different FP bio-oils, clearly show that the aging process occurs in two distinct stages: a sharp drop in carbonyl content during the first 4 hours of aging, followed by a nearly constant carbonyl content during subsequent aging from 4 hours out to 72 hours.

This indicates that there is a significant fraction of the carbonyl content that is unreactive. Previous research^27^ has assumed that this unreactive carbonyl content is in the 1.5 mol kg^−1^ range following a week at 70–86 °C aging. These measurements, however, were done using an older method of carbonyl determination that has been shown to result in incomplete oximation of the carbonyls present in the bio-oil, and therefore yield erroneously low carbonyl contents.^29^ In Fig. 7–10, unreactive carbonyl contents are in the range of 2–3 mol kg^−1^. It is likely that reactive carbonyls are reactive aldehyde species formed from the dehydration of sugars. These reactive aldehydes are actively involved in the resol-like reactions of the aging process, and account for *ca.* 60% of the carbonyls quantified. Once the reactive carbonyls are depleted from the aging process, the remaining unreactive carbonyls remain constant upon further heat treatment. The unreactive carbonyls are likely ketone groups, as the aldehydic groups have been completely consumed.^8^ As seen in Fig. 7–10, reactive carbonyls are depleted by 24 hours of accelerated aging at 80 °C.

It is worth noting that viscosity continues to increase at longer aging times, after reactive carbonyls are depleted, see Fig. 3–5. The large error in the viscosity measurement makes it difficult to clearly see a trend of increasing viscosity, but generally, viscosity appears to slightly increase beyond 24 hours of aging at 80 °C. We propose that any additional increase in viscosity after reactive carbonyls are depleted is due to internal crosslinking reactions, which does not appear to involve further consumption of the unreactive carbonyls.^8^ Additionally, for some samples, it does appear that the viscosity increase significantly slows down after reactive carbonyls are depleted; see Fig. 5.

While the previously used aging protocol (80 °C for 24 hours) is too severe to correlate to room temperature aging, it can be used for the measurement of unreactive carbonyl content, as reactive carbonyls have been fully depleted by 24 hours for all samples studied. For this reason, we propose that accelerated aging at 80 °C for 24 hours be used for the measurement of total unreactive carbonyl content.

### Low temperature storage

In the bio-oil community, aging at room temperature has been well known for several decades. Common practice has been to preserve samples by storage at temperatures well below room temperature. Here, we show that aging still proceeds at these lower temperatures, as evidenced by the steady decrease in carbonyl content at both 9 °C and −17 °C in Fig. 7–9. Storage at both 9 °C and −17 °C results in *ca.* 10% reduction in carbonyl content after 6 months. At long times, out to 3.4 years of storage, carbonyl contents are *ca.* 20% lower than for the fresh samples for Blend1, Oak, and Pine samples. This is a significant loss of carbonyl content for samples stored in the freezer, and clearly shows that the bio-oil aging process is suppressed, but not stopped, at lower temperatures. Additionally, there is a clear correlation to temperature, with the coldest storage condition (−17 °C) resulting in more stable carbonyl contents than storage at a milder condition (9 °C). These results show that a fresh bio-oil sample cannot be preserved, even with storage at low temperatures. The aging processes occurring in the bio-oil begin immediately upon condensation of the pyrolysis vapors and will continue to occur with time. This has large implications for the archiving of samples, and for the timeline of when bio-oils samples are analyzed. Researchers need to keep in mind that a FP bio-oil sample will be continually changing immediately after production, regardless of storage condition.

## Conclusions

Bio-oils change significantly with aging, both in terms of physical properties as well as chemical composition. Due to this, a reliable accelerating aging test is needed, and it could be used to gauge how a sample has changed, or will change, with varying times of storage at room temperature. This type of information would be very useful in many different industrial processing strategies and will inform decision making around both the processability of the bio-oil at different storage times, as well as how to run the process itself. Bio-oil aging at room temperature results in an increase in viscosity, which has traditionally been used to track bio-oil aging. However, there are several problems with using viscosity to track aging, including a high variability in the measurement, and the fact that viscosity cannot be measured for bio-oils that have phase separated during aging, which is a common occurrence. Here, we have shown that total carbonyl content is a much better metric for tracking aging, as it has a significantly lower measurement variability, and can also be applied to samples that have phase separated. Furthermore, the carbonyl titration method employed has been shown to be very robust, and has recently been standardized as ASTM E3146.

By comparing different accelerated aging protocols (at both 40 °C and 80 °C) and comparing their results to actual room temperature storage (for over 3 years) of a suite of different fast pyrolysis bio-oils, we have developed a new accelerated aging protocol for bio-oils. Also, we have shown that a widely used accelerated aging protocol (24 hours at 80 °C) is too severe a heat treatment, and results in more extensive aging of all samples than was seen at 3.4 years of storage at room temperature (which was the maximum period studied). Here, we propose a new aging test of 2 hours at 80 °C, with carbonyl measurement before and after heat treatment. This heat treatment correlates to 1–3 months of storage at room temperature, a reasonable amount of time that a sample would likely be stored at room temperature. Finally, the significant reduction in carbonyl content for samples in cold storage (even at −17 °C) was an unexpected result. This shows that storage of samples for archival purposes is problematic, and both the physical and chemical properties of a bio-oil cannot be preserved in cold storage. While aging in cold storage would not likely effect industrial bio-oil processing, this is important for the research community, where archival of samples in cold storage is commonly employed.

## Conflicts of interest

There are no conflicts to declare.

## Supplementary Material
